# Nitric Oxide Synthase Expression in Endometrium During Physiological Cycle

**DOI:** 10.33549/physiolres.935735

**Published:** 2025-12-01

**Authors:** Tereza KURACINOVA, Miriam BOLLOVA, David KOCAN, Kristina MIKUS KURACINOVA, Andrea JANEGOVA, Pavol JANEGA

**Affiliations:** 1 Institute of Pathological Anatomy, Faculty of Medicine, Comenius University in Bratislava, Slovak republic; 2 Institute of Normal and Pathological Physiology, Centre of Experimental Medicine, Slovak Academy of Sciences, Bratislava, Slovak republic; 3 Medirex Group Academy, n.p.o., Nitra, Slovak Republic

**Keywords:** Endometrium, Menstrual Cycle, Nitric Oxide Synthase, Nitric Oxide, Natural Killer Cells

## Abstract

The human endometrium undergoes dynamic hormonal and structural changes throughout the menstrual cycle. Their aim is to create an environment essential for embryo implantation. Successful implantation depends on the proper composition of the endometrial microenvironment, including cytokine synthesis and local immune responses. During the first trimester, uterine natural killer (uNK) cells play a key role in regulating trophoblast invasion, vascular remodelling, and establishing embryo tolerance, with nitric oxide (NO) also contributing to these processes. The study aimed to evaluate the expression patterns of NOS2 and NOS3 and their relationship to the infiltration of endometrium by uterine natural killer (uNK) cells during different menstrual phases. The endometrial tissue samples representing proliferative, early secretory, late secretory, menstrual, and hypersecretory phases were analysed by immunohistochemistry and fluorescence microscopy. NOS2 and NOS3 showed distinct cyclic patterns. NOS3 expression peaked in the early secretory phase, supporting tissue remodelling, while NOS2 expression increased progressively, reaching its maximum in the late secretory and menstrual phases. The number of uNK cells paralleled NOS2 expression, with a positive correlation suggesting a possible NO-related immunomodulatory mechanism. Elevated NOS2 expression and increased clustering of CD56^+^ uNK cells were observed in some cases of proliferative endometrium, possibly reflecting phase-inappropriate immune activation. These findings indicate that NOS activity and uNK cell dynamics may jointly contribute to the cyclic regulation of the endometrial microenvironment. Understanding NOS regulation and its hormonal and immune interactions may offer new insights into implantation mechanisms.

## Introduction

The human endometrium is a tissue influenced by dynamic hormonal fluctuations throughout the menstrual cycle. Variations in oestrogen and progesterone levels are responsible for the phases of proliferation, secretion, and shedding, followed by tissue regeneration from the basal layer. These changes involve a functional transformation of the endometrium, which is essential for creating an optimal environment for embryo implantation.

Nitric oxide (NO), a small and highly reactive gaseous molecule, acts as a key mediator in many biological processes, including regulating vascular tone, neural communication, immune response, and tissue homeostasis. Current knowledge shows that it plays a complex regulatory role in the physiological endometrium and is considered one of the essential paracrine mediators throughout a physiological menstrual cycle [1,2]. Because NO is highly unstable and degrades quickly, its effects are limited to the site of production, where it facilitates short-range signalling. Detecting NO directly in tissues is challenging; thus, studies often measure NOS enzyme expression and activity or evaluate its stable byproducts as indirect indicators of NO production. NO levels in endometrial fluid rise from the late proliferative phase through the secretory phase, reaching their peak during menstruation [3].

Three isoforms of NOS are identified, each varying in regulation and physiological context. In the endometrium, NO is mainly produced by inducible (NOS2) and endothelial nitric oxide synthase (NOS3). In physiological endometrial tissue, NOS3 is the dominant isoform, consistently found in the glandular epithelium and microvascular endothelium. The level of NOS3 expression is higher during the secretory phase than in the proliferative phase of the menstrual cycle. The activity of NOS2 also progressively increases and reaches its peak during the menstrual phase [4]. The precise regulation of these enzymes, along with modifications in their synthesis and localised activity, is crucial for maintaining optimal nitric oxide levels required for various cellular and organ-specific processes, especially in endometrial physiology. [5,6].

The production of NO in the endometrium is influenced by hormonal fluctuations. It was demonstrated that oestrogen upregulates NO production [7]. Conversely, the impact of progesterone is not entirely clear. While some studies propose that it may have an opposing effect on NO production, other findings report elevated NO levels during the luteal phase. It seems that physiological concentrations of progesterone might enhance the stimulatory effect of oestrogen on NO synthesis under certain physiological conditions [7–9].

The production of NO in the endometrium plays a crucial role in embryo implantation and supports successful pregnancy. Inhibition of nitric oxide synthase by L-NAME in animal studies led to implantation failure, decreased uterine permeability, and impaired embryo development. Co-administration of a nitric oxide donor reversed these effects [10]. NO regulates endometrial blood flow, especially during the secretory phase. It also participates in the vascular remodelling of the endometrium [11,12]. During the secretory phase, NO also inhibits platelet aggregation and suppresses myometrial contractility, contributing to uterine quiescence [13,14]. Disruption of NO signalling may therefore be linked to impaired implantation and various reproductive disorders.

It is well established that NO modulates immune system activity. A finely balanced immune environment is crucial for establishing tissue tolerance towards the developing fetus and ensuring successful implantation. The main immune cell populations involved in this process include uterine natural killer (uNK) cells, macrophages, dendritic cells, regulatory T lymphocytes (Tregs), and other T-cell subpopulations. During the first trimester, uNK cells make up more than half of the immune cell population in the decidua. These cells orchestrate the local immune response, regulate trophoblast invasion and vascular remodelling, and are essential for maintaining a receptive and embryo-tolerant uterine microenvironment [15,16].

Several aspects remain insufficiently understood, especially concerning the practical application of diagnostic testing and therapeutic approaches aimed at modulating NO production in gynaecology. Although the role of NO in mediating physiological changes within the endometrium has been described, the potential for personalised NO modulation as a therapeutic target in the treatment of infertility or recurrent implantation failure remains unexplored. The aim of this study was to evaluate the variability of NO synthase expression throughout the physiological menstrual cycle and to identify the mechanism of its potential impact on the regulation of successful implantation. A deeper understanding of NOS regulation and its interactions with hormonal and immune factors is a crucial step towards developing future personalised therapeutic strategies.

## Methods

A total of 50 endometrial tissue samples from women aged 30 to 45 were included in the study. The samples came from the archive of the Institute of Pathological Anatomy, Faculty of Medicine, Comenius University in Bratislava, and from the Medixbank biobank, Medirex Group Academy, n.p.o. All samples were obtained from women who had undergone uterine curettage for diagnostic or therapeutic reasons. Only specimens with histologically confirmed physiological endometrium at various menstrual cycle stages were included. Patients with a current or prior diagnosis of malignant disease were excluded. The study group consisted of 50 cases, 10 cases each of proliferative endometrium, early secretory endometrium, late secretory endometrium, menstrual endometrium, and hypersecretory endometrium with stromal decidualization. The research was approved by the Ethics Committee of the Faculty of Medicine, Comenius University, and the University Hospital Bratislava and the Ethics Committee of the Bratislava Self-Government Region as part of the Biomedires 2 project.

For immunohistochemical and fluorescence analyses, 3 μm thick formalin-fixed, paraffin-embedded tissue sections were prepared on glass slides. After standard deparaffinization, antigen retrieval was performed in citrate buffer (10 mM, pH 6,0, with Tween) using the PT Link system (Agilent, Santa Clara, California, USA) for NOS2 and NOS3 antibodies, and in TRIS-EDTA buffer (10 mM TRIS, 1 mM EDTA, pH 9.0, with Tween) using a pressure cooker for 20 minutes for CD56. The following primary antibodies were used: NOS2 (rabbit polyclonal, Merck Millipore ABN26, dilution 1:100), NOS3 (mouse monoclonal, Bios bsm-33176M, dilution 1:100), and CD56 (Agilent, clone 123C3, ready-to-use). Detection of NOS2 and NOS3 was carried out using fluorescent labelling with VectaFluor™ Excel Amplified Anti-Mouse IgG, DyLight® 488, and VectaFluor™ Excel Amplified Anti-Rabbit IgG, DyLight® 594 (Burlingame, California, USA). For CD56, enzyme-based immunohistochemistry was performed using N-Histofine® Simple Stain™ MAX PO (Nichirei Biosciences Inc., Tokyo, Japan), followed by DAB as the chromogen for visualisation.

All slides were examined using a Nikon Eclipse Ci fluorescence microscope (Nikon, Tokyo, Japan). The fluorescence intensity of NOS2 and NOS3 was measured morphometrically with ImageJ software, Fiji version, by quantifying the total fluorescence signal within the epithelial cells. For each case, five independent measurements were performed. The presence of CD56-positive uterine natural killer (uNK) cells was also assessed morphometrically with the same software and expressed as the percentage of CD56-positive cells relative to the total number of stromal cells in the evaluated area.

Statistical analysis was performed using GraphPad Prism version 10.6.1 (GraphPad Software, San Diego, CA, USA). Data distribution was evaluated with the D’Agostino and Pearson test for normality. For data following a Gaussian distribution, one-way ANOVA was performed, followed by Tukey’s multiple comparisons test. When the data did not meet normality criteria, the non-parametric Kruskal–Wallis test was used. The correlation between NOS2 expression and the proportion of CD56-positive NK cells was assessed using Spearman’s rank correlation coefficient (Spearman r). The results are expressed as mean ± standard deviation (SD). The language of the manuscript was reviewed with Grammarly software.

## Results

**Fig. 1:**
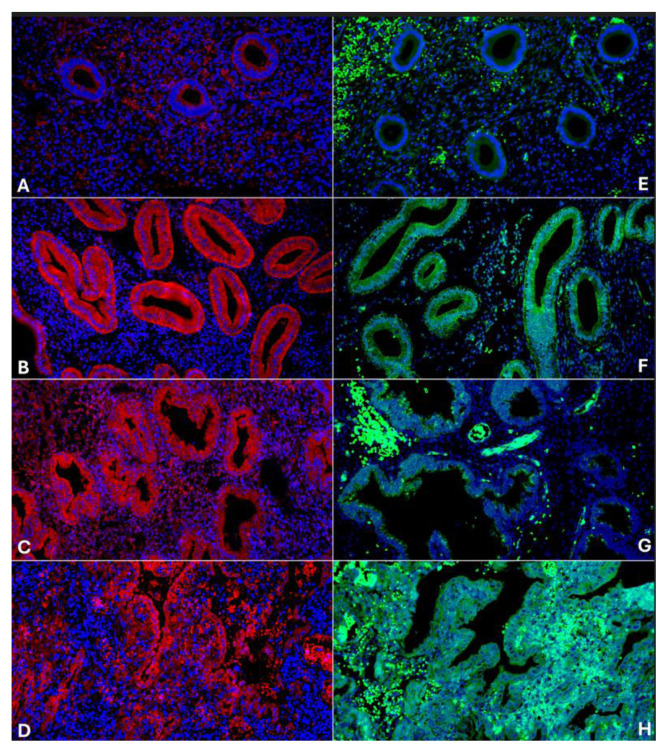
Endometrial expression of NOS2 and NOS3 across menstrual cycle phases and hypersecretory endometrium. Diffuse cytoplasmic NOS2 positivity is observed in the glandular epithelium across all examined stages, with the lowest intensity seen in most cases of proliferative endometrium (**A**). The signal increases in the early (**B**) and late secretory phases (**C**), and is also elevated in hypersecretory endometrium (**D**). NOS3 expression is minimal in proliferative endometrium (**E**), reaches its peak during the early secretory phase (**F**), and subsequently declines in the late secretory phase (**G**), notable expression persists in hypersecretory endometrium (**H**). NOS2, IF DyLight 594 (red signal), 200x; E–H: NOS3, IF DyLight 488 (green signal), 200×.

A consistent diffuse cytoplasmic positivity of NOS2 in endometrial glands was observed across all examined cases. The lowest positivity was found in 7 out of 10 cases of proliferative endometrium (25.48 ± 4.72 AU). Positivity significantly increased during the early secretory phase (38.12 ± 6.35 AU) and reached its peak in the late secretory endometrium (47.16 ± 8.79 AU) (Figs 1 and 3). A slight but significant decrease in NOS2 positivity occurred in the menstrual phase (39.88 ± 5.36 AU) and in hypersecretory endometrium with stromal decidualization (37.60 ± 5.06 AU). An interesting observation was the presence of high NOS2 expression in 3 out of 10 cases of proliferative endometrium (51.78 ± 7.88 AU), which did not significantly differ from that seen in the late secretory phase. Stromal NOS2 expression showed no marked differences among the groups, with mean values ranging from 15.55 ± 5.16 AU in the proliferative phase to 20.83 ± 3.43 AU in the menstrual phase.

The lowest NOS3 positivity in endometrial glands was observed during the proliferative phase (19.91 ± 1.09 AU), followed by a significant increase reaching its peak in the early secretory phase (25.31 ± 1.56 AU). A notable decline then occurred in the late secretory phase (20.98 ± 2.02 AU) (Figs 1 and 3). A slight decrease in NOS3 positivity was also seen in the menstrual phase (18.75 ± 2.71 AU), where it was no longer significantly different from the proliferative phase. Additionally, high NOS3 expression was detected in the hypersecretory endometrium with stromal decidualization (24.67 ± 4.11 AU).

**Fig. 2:**
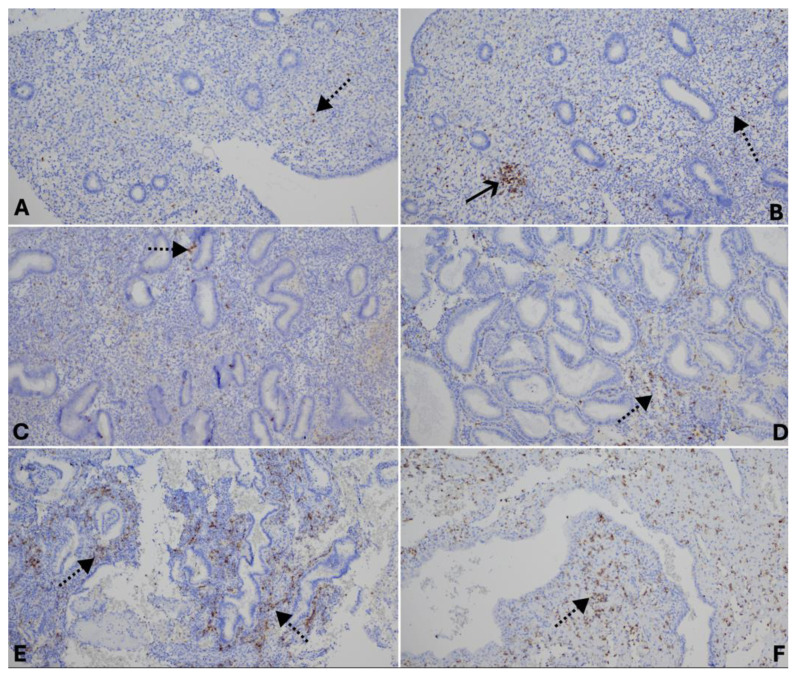
Distribution of CD56^+^ uterine natural killer (uNK) cells across menstrual cycle phases and in hypersecretory endometrium. Proliferative (**A**), early secretory (**C**), and late secretory endometrium (**D**) show a gradual increase in uNK cell numbers (dotted arrows). In some cases of proliferative endometrium (**B**), unusually high numbers of uNK cells forming clusters are also observed (solid arrows). Menstrual endometrium (**E**) and hypersecretory endometrium with stromal decidualization (**F**) demonstrate the highest density of CD56^+^ uNK cells. CD56, IHC-Px, 200×.

**Fig. 3:**
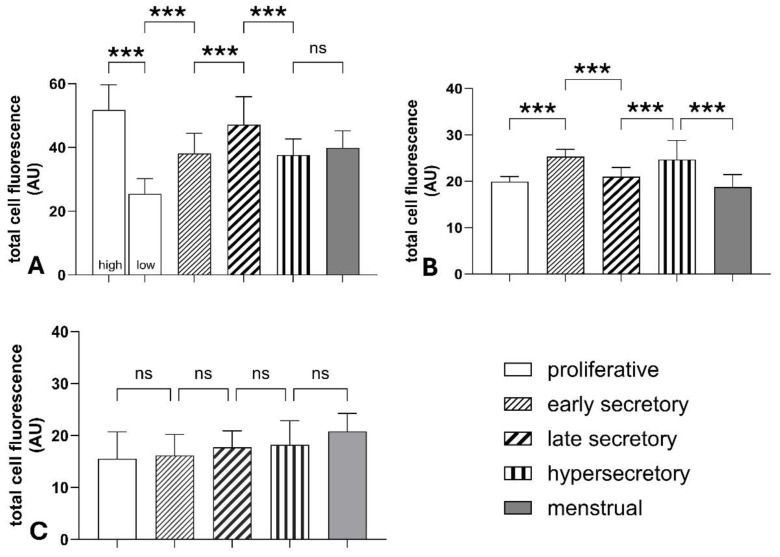
Analysis of NOS2 and NOS3 expression in endometrial glands and stroma across menstrual cycle phases and in hypersecretory endometrium. (**A**) NOS2 expression in glandular epithelium exhibits significant variability, with the lowest levels in proliferative endometrium (low) and a gradual increase towards the secretory phases. In occasional cases of proliferative endometrium (high), unusually high NOS2 expression was observed. (**B**) NOS3 expression in glandular epithelium rises significantly in the early secretory phase compared to the proliferative phase and decreases in the late secretory and menstrual phases. (**C**) NOS2 expression in the stroma remains relatively stable, with no significant differences among the examined groups. Total cell fluorescence values are presented as mean ± SD in arbitrary units (AU). Statistical significance: ***p < 0.001, ns = non-significant.

**Fig. 4:**
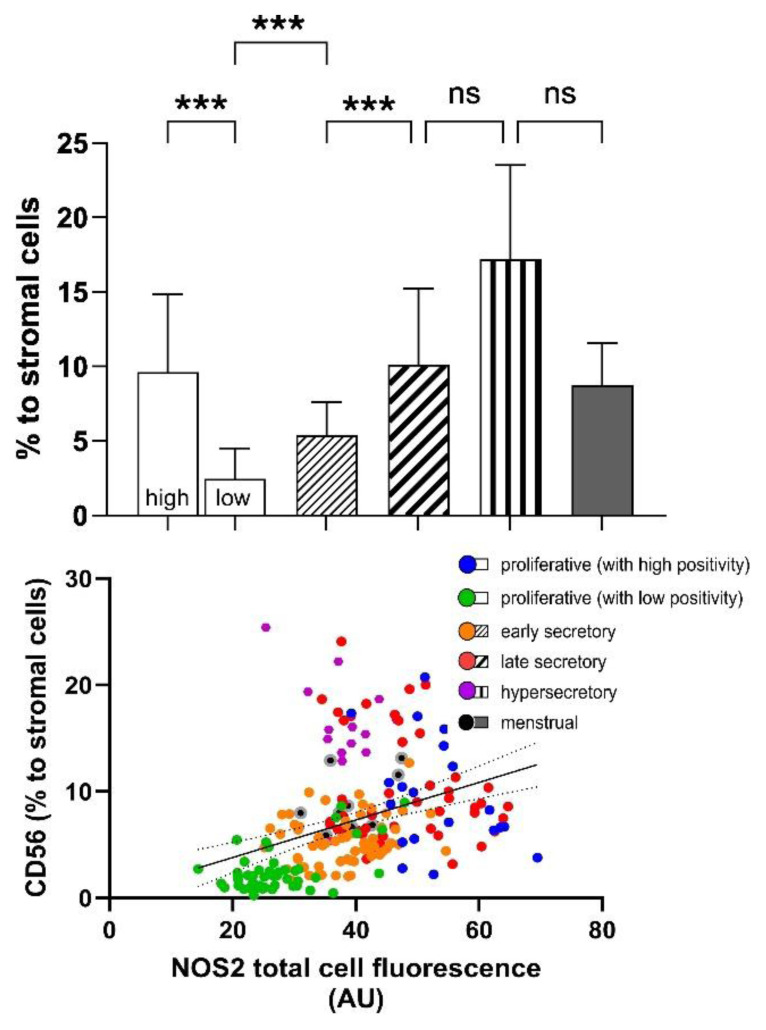
Association between NOS2 expression and the proportion of CD56^+^ uterine natural killer (uNK) cells. The proportion of CD56^+^ uNK cells shows marked variability among the groups. The lowest values were observed in most proliferative endometrium cases, whereas proliferative samples with unusually high NOS2 expression showed increased uNK cell infiltration. A progressive rise was evident through the early and late secretory phases. No significant differences were found among the late secretory, menstrual and hypersecretory endometrium. Data are presented as mean ± SD. Statistical significance: ***p < 0.001, ns = non-significant. AU, arbitrary units.

A similar trend was observed in the number of CD56^+^ uterine natural killer (uNK) cells. Their distribution correlated with the level of NOS2 positivity, reaching its minimum in the proliferative endometrium (2.45 ± 2.05 %) and gradually increasing in the early (5.37 ± 2.23 %) and late secretory phases (10.12 ± 5.10 %). The number of uNK cells did not differ significantly in the menstrual endometrium (8.73 ± 2.81 %) and hypersecretory endometrium with stromal decidualization (17.19 ± 6.32 %) compared to the late secretory phase (Fig. 2). Interestingly, a high number of uNK cells (9.61 ± 5.22 %) was also observed in cases of proliferative endometrium with high NOS2 expression, often forming clusters of CD56^+^ cells. The number of infiltrating uNK cells correlated with the intensity of NOS2 positivity in the surrounding endometrial cells (Fig. 4).

## Discussion

The present study demonstrated that NOS2 and NOS3 expressions vary throughout the menstrual cycle, although we observed interindividual variability in the levels of expression. A gradual increase is noted towards the subsequent phases. The changes involve both inducible NOS2 and endothelial NOS3, indicating that both enzymes may be significant sources of NO within the endometrial tissue. The released NO contributes to preparing an adequate environment for embryo implantation; it participates in controlling immune tolerance, blood vessel remodelling, and trophoblast invasion. Dysregulation of NO was associated with implantation disorders in other studies. Targeted modulation of nitric oxide proves effective in animal models. The nitroglycerin treatment significantly enhanced sub-endometrial blood flow in women experiencing unexplained infertility. During the secretory phase, the released NO and other cytokines promote vascular transformation and support embryo adhesion [2,17].

The increased expression of NOS3 highlights its potential role in establishing endometrial receptivity and supporting adequate uterine blood flow necessary for successful implantation and early embryonic development. The presented results showed that NOS3 expression peaked during the early secretory phase and then declined towards the late secretory and menstrual phases. These findings agree with Khorram *et al.*, who found the highest NOS3 expression in the mid-secretory stage. This pattern supports that NOS3 may play a role in early secretory endometrial function, possibly related to vascular regulation and preparation for implantation [4,18].

In contrast, in the present study, NOS2 expression gradually increased throughout the menstrual cycle, reaching its peak during the late secretory and menstruation phases. Higher NOS2 expression in the endometrium during the secretory phase was also described in other studies. In animal experiments, both NOS2 and NOS3 are highly upregulated at implantation sites and have physiological functions during implantation and early pregnancy, with NOS2 potentially becoming the dominant NOS isoform. Additionally, stromal decidualization is generally accompanied by the upregulation of NOS2 [2]. Concurrently, the continuous rise of NOS2 indicates an increasing role in local inflammatory and tissue remodelling processes that occur before menstruation, and the high NO levels in this phase may exert cytotoxic effects, participating in the process of menstrual disintegration [12].

The induction of NOS2 in the endometrium during the menstrual phase plays at least a partial role in the initiation and maintenance of uterine bleeding. During menstruation, local NO production acts as a potent vasodilator and regulates platelet activity by inhibiting platelet aggregation. When the endometrial blood vessels are disrupted, platelets adhere to exposed subendothelial structures to form a haemostatic plug. However, NO modulates this process at multiple stages by inhibiting platelet adhesion and aggregation. Consequently, NO helps balance vasoconstriction, coagulation, and tissue breakdown, linking vascular and haemostatic mechanisms that underpin normal menstrual bleeding [2,12].

In endometrial tissue, CD56^+^ uterine natural killer (uNK) cells are observed throughout all phases of the menstrual cycle. Their proportion is lowest in the proliferative phase (2.45% of all stromal cells) and gradually increases during the early (5.37%) and late secretory phases (10.12%). The presence of uNK cells is considered an important factor in establishing immune tolerance towards the implanting embryo. During the window of implantation, uNK cells form the largest leukocyte population in the endometrium [19]. The percentage of uNK cells observed in our study aligns well with the reference limits reported by Yu *et al.* [18], who described that uNK cells represent 1.8–8.7% of all stromal cells during the mid-luteal phase. Differences observed in our study may relate to variations in the methodological approach, particularly in the computer-based quantification of the evaluated areas. This finding emphasises the importance of standardising the assessment of uNK cells, especially if these measurements are to be used for diagnostic or prognostic purposes in reproductive medicine [20].

A high level of uNK cells was also observed in hypersecretory endometrium with stromal decidualization. This may focus on the role that uNK cells play in regulating the immune microenvironment during the early stages of pregnancy. On the other hand, it remains unclear whether such significant infiltration associated with high NOS2 and NOS3 expression reflects a physiological process supporting immunological tolerance or, conversely, contributes to oxidative stress and a pro-inflammatory phenotype potentially associated with pregnancy failure. The samples evaluated in our study, which show decidualization, are incidental findings and therefore cannot be considered representative of physiological pregnancy. It is well described that the physiological function of uNK cells depends on their proper regulation, and excessive activity can lead to disrupted implantation [19].

In our study, we demonstrate that the increase in NOS2 expression correlates with a higher number of uNK cells in the endometrium. This relationship suggests a possible link between NO production and the recruitment or activation of immune cells during the menstrual cycle. Although other studies have not documented such a correlation, it is known that NO plays a significant immunomodulatory role, participating in the differentiation process of uNK cells and influencing their function. The endogenous production of NO contributes importantly to successful pregnancy [17,21].

An intriguing observation was the presence of high NOS2 expression and increased uNK cell counts in some cases of proliferative endometrium, at levels similar to those typically seen in the late secretory phase. Although clinical correlation is unavailable, such phase-inappropriate patterns could signal a pathological process and could impact reproductive outcomes. Elevated uNK cell density combined with NOS2 upregulation may contribute to implantation issues. An optimal uNK level is essential for early placental development, and dysregulation has been associated with reproductive disorders [20]. Simultaneously, NOS2-derived NO influences endometrial receptivity, while excessive NO can promote nitrosative stress and a pro-inflammatory phenotype [16]. We hypothesise that concurrent excessive increases in CD56^+^ uNK activity and NOS2 expression might intensify local inflammation and oxidative stress, upsetting the immune–vascular balance necessary for successful endometrial development.

The present study demonstrated that physiological cyclic changes in the endometrium are associated with variations in nitric oxide synthases expression. These fluctuations may affect the overall balance of the endometrial microenvironment, thereby influencing implantation potential and pregnancy success. However, the precise mechanisms behind these processes remain unclear. Future research should investigate the role of nitric oxide signalling in infertility and examine its potential for therapeutic modulation.
